# Correct administration aid for oral liquid medicines: Is a household spoon the right choice?

**DOI:** 10.3389/fpubh.2023.1084667

**Published:** 2023-02-20

**Authors:** Eman Younas, Moomna Fatima, Ayesha Alvina, Hafiz Awais Nawaz, Syed Muneeb Anjum, Muhammad Usman, Mehak Pervaiz, Amara Shabbir, Huma Rasheed

**Affiliations:** Institute of Pharmaceutical Sciences, University of Veterinary and Animal Sciences, Lahore, Pakistan

**Keywords:** oral liquid medication, pediatrics, medication safety, rational use of medicines, household spoon, administration aid

## Abstract

**Background:**

Correct medicine dosing is an important component in the safe and effective delivery of medicines, particularly for the pediatric population. However, there is a scarcity of public campaigns on the correct administration and choice of dosing aids for oral liquid dosage form in many countries, leading to medicine safety issues and therapeutic failures.

**Methods:**

The study targeted the assessment of the knowledge and practice of university students. It utilizes pre- and post-intervention surveys administered through google forms as a survey tool during online zoom and in-person sessions. The intervention included a short video presentation detailing the selection and use of medicine spoons and other aids for the administration of oral liquid dosage. The Fischer Exact test was used to assess the pre- and post-test shift of responses.

**Results:**

Nine-degree programs were engaged in the activity, and 108 students attended this health awareness activity after obtaining formal consent. A significant decline (CI = 95%, ^****^p-value < 0.05) in the choice of selecting tablespoon and a shift to a low-volume spoon, as well as rejection of an entire variety of household spoons, were observed. A significant improvement in the correct naming of spoons, the meaning of the abbreviation “tsp,” and the correct volume of a standard teaspoon were also observed with a *p*-value of <0.001.

**Conclusion:**

A deficit in the knowledge of the proper use of measuring devices for oral liquid medicines in the educated population was observed, which can be enhanced through simple tools like short video presentations and awareness seminars.

## Public interest summary

One of the key responsibilities of the healthcare system is to ensure medication safety. Various factors contribute to ensuring this, including but not limited to packaging, labeling, dosing, consumption, and marketing. This study assesses the correctness of household spoons as dosing and administration aid for oral liquid dosage forms among university students using a short video presentation as an intervention tool. The message in the video focused on disseminating the risks involved with the use of a common household spoon. Assessments were made before and after video presentation and the data were analyzed for the knowledge of respondents and its shift with the use of intervention.

The revealed that the digitally enabled population of university students were receptive to short video presentation highlighting the avoidable risks in medication management.

## Introduction

Oral liquid pharmaceutical dosage forms are widely accepted and used, especially in pediatric and geriatric care. Their safety and effectiveness depend on the right dosing measurement (1). Dose measurement is a common step prone to error with drug administration (2–4), especially with liquid formulations such as syrups, suspensions, elixirs, linctus, and solutions (3) unless the packaging is supplemented with the correct administration containers. These dosage forms include common therapeutic categories, for instance, antipyretics, analgesics, anti-cough, and flu remedies, antibiotics, laxatives, and multivitamins. Most people tend to use household spoons for oral dosing (5). Most commonly available household spoons include 5 ml teaspoons, 10 mL dessert-spoon, 15 mL soup spoons, and 15 mL tablespoons, which vary greatly in their design and volumes that they can accommodate (1).

The variation in shapes, sizes, forms, and make of these household spoons leads to dosing errors (6). The American Pharmaceutical Association in 1902 and American Medical Association in 1903 defined the “standard teaspoonful” as 5 mL (7). The American Academy of Pediatrics (AAP) Committee on Drugs reported that, when liquid medicine is not provided with a teaspoon, 75–80% of people use a household teaspoon as an alternative to standard teaspoons (8). One study from Israel reported that 80% of children are given medications by a household teaspoon (9). A similar report from Minnesota, United States, stated that a household teaspoon was the device most frequently used by 73% of the population for measuring liquid medications (5).

Dosing and administering medication for the pediatric population is even more difficult as compared to the adult population as they need to be adjusted according to age and body weight. As a result, children are more vulnerable to dosing errors (2, 10, 11). Calibrated devices such as dosing cups, oral droppers, and oral syringes have been recommended to measure and administer liquid medication to the pediatric population. However, the oral syringe is found to be the most convenient and accurate dosing device (12).

Various studies showed that the most commonly used medicinal aids for oral liquid dosing are cylindrical spoons, dosing cups, droppers, teaspoons, oral syringes, and spoons or syringes with bottle adapters (6, 12). Dosing cups, droppers, and household spoons are mostly used for the measurement of oral liquid dosing at home (12). Spoons and dosing cups are at a higher disadvantage for doses that are not multiples of 5, like 0.5 or 3.5 mL and are not measurable with a cup marked with 5, 10, 15, and 20 mL (13). Oral syringes account for the least percentage of imprecise dosing (12). A spoon or syringe with a bottle adapter and dispensing bottle also provided accurate results (14–16).

This study aimed to evaluate the pre- and post-intervention knowledge of university students about the choice of household spoons and oral liquid dosing aids in Pakistan.

## Methods

Pre- and post-awareness surveys were conducted using predesigned questionnaires on undergraduate students of the University of Veterinary and Animal Sciences (UVASs), Lahore, who have undergone at least 1 year of university education.

**Figure 1:**
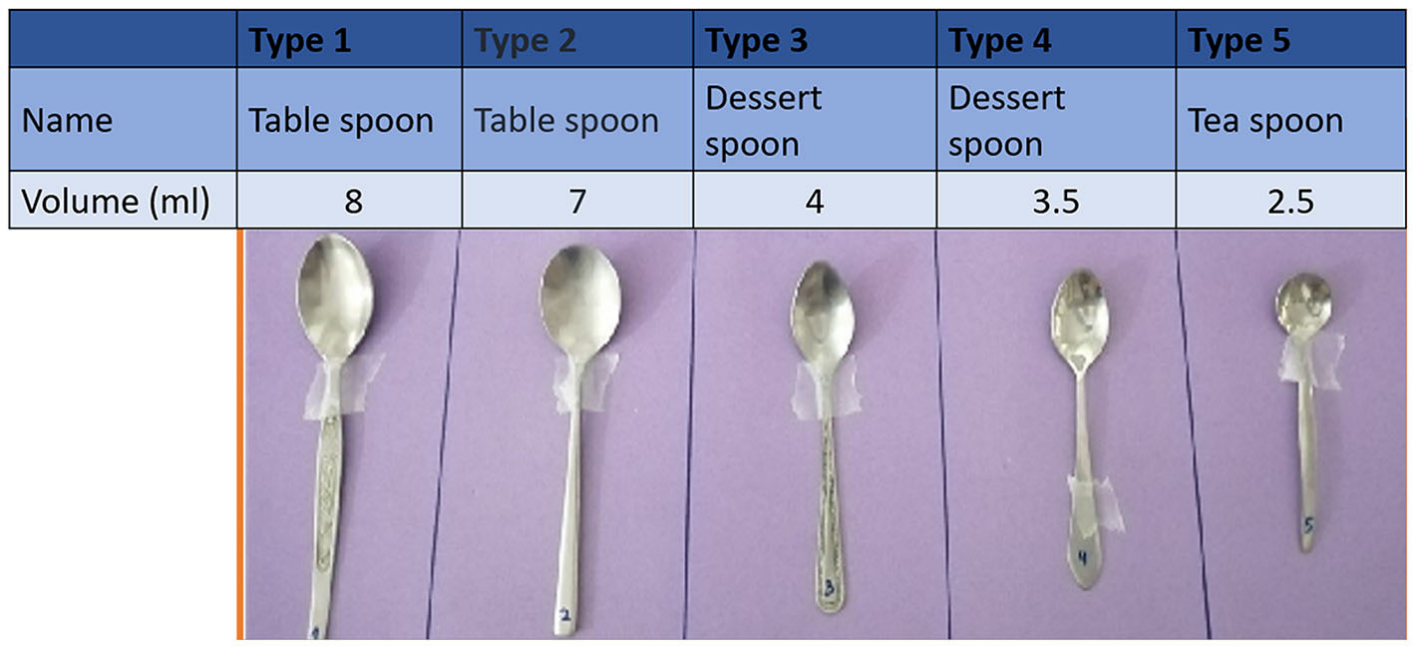
Chart One: the selection of a spoon for the administration of medicine from household spoons, along with actual measurements.

**Figure 2:**
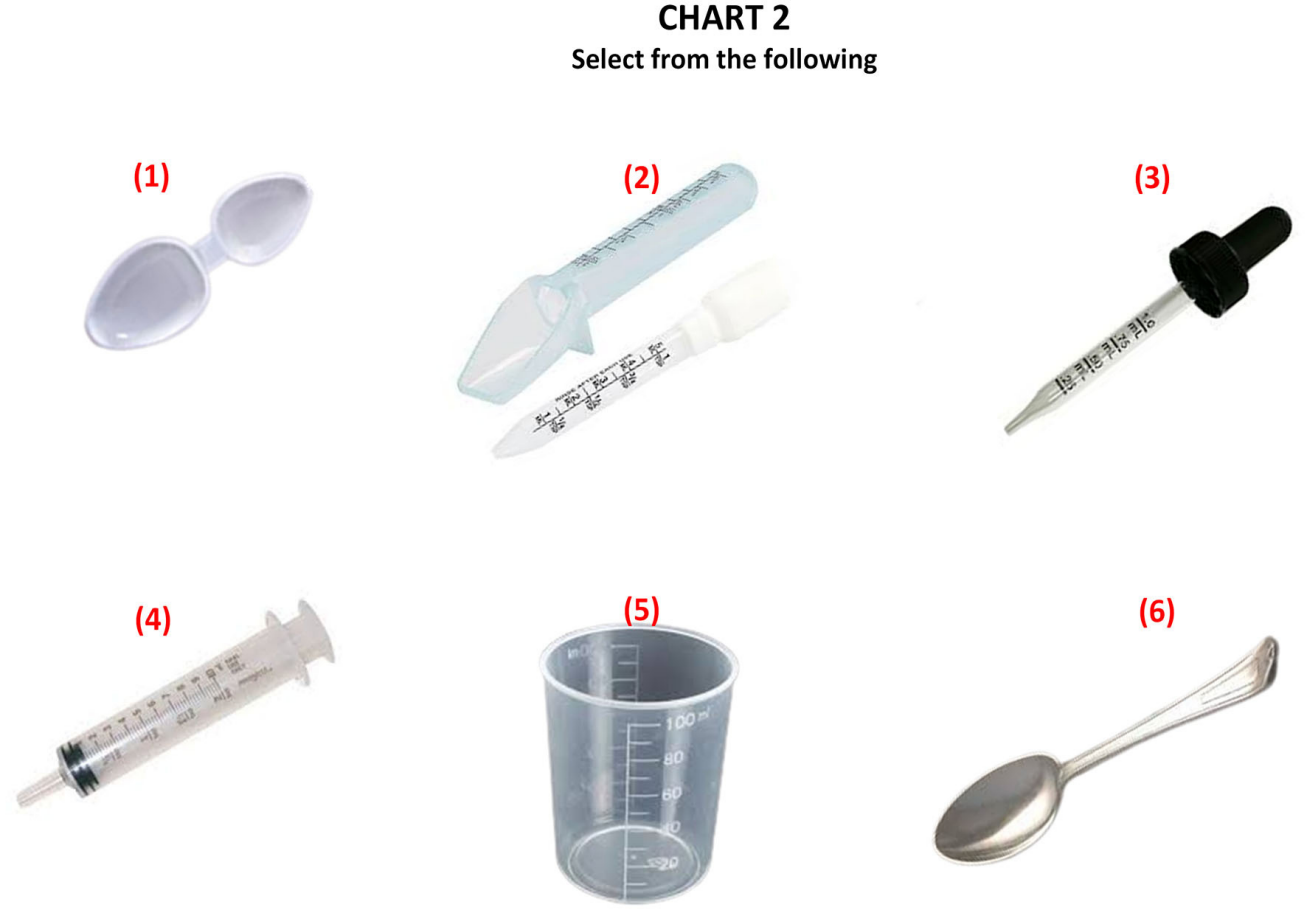
Different oral liquid medicine administration aids.

A sample size of 108 (12% of 908) students was targeted using convenient sampling. Students from nine different degree programs were invited in person by meeting post-class for volunteer participation to attend the public health awareness session. Interested students were placed in a WhatsApp group for efficient communication. A convenient time was fixed for the session in which students were connected on zoom call. They were provided with a link to a Google form for a pre-intervention survey which was followed by the video presentation. Similarly, the post-intervention form was shared in a second zoom call after the video presentation. The questionnaires (Supplementary material 1) and video presentation included the following segments:

- Assessment of health literacy of university students regarding the use of medicine spoon.
- Educating students about problems associated with the use of household spoons for use and the choice of aids for administering oral liquid medicines (Figure 1).
- Raising awareness about different choices of tools for oral liquid medicines and the importance of correct dosing (Figure 2).
- Propose recommendations to avoid dosing errors.
- To develop skills in health promotion and health education among university students working under the core team of female students.

The key component of the study was two charts. The first chart displayed the photographs of five differently sized household spoons (Figure 1). The spoon included two different designs of tablespoon/dinner spoons (I and II), two teaspoons (III and IV), and a sugar spoon (V). The spoons were arranged in the order of decreasing sizes. They were calibrated using a 5-mL syringe and labeled with the exact volume in milliliters, and this information was shared in the awareness session. The respondents were also briefed on how they can do a small experiment at home to know the volume of the household teaspoon to calibrate it, in case it is inevitable to use. The other chart displayed the photographs of six different tools for oral liquid medication administration, including (1) a medicine spoon, (2) a medicine spoon with graduated tubing, (3) a graduated dropper, (4) an oral syringe, (5) a graduated cup/beaker, and (6) a household teaspoon (Figure 2).

Ethical approval was received from the Institutional Review Board of The University of Veterinary and Animal Sciences under the letter No. 174/IRC/BMR, dated: 03.03.2022, to carry out this study. Training of the trainers was conducted by the principal investigator on a team of four researchers who carried out surveys and awareness sessions with the students of different degree programs. A pilot was first performed by four lead trainers on the Doctor of Pharmacy (Pharm.D) student's batch through hybrid zoom and in-person sessions. After this session, necessary changes were made to the survey form and presentation. The survey was divided into two phases. In the first phase, a pre-intervention survey was conducted to analyze the knowledge of the students of Pharm.D about the use of household spoons and other medical aids for oral dosing. Subsequently, an awareness training session was conducted using a video recording on zoom to inform the respondents about the right choices for oral dosing, the consequences of using the household spoon and wrong dosing, and the use of graduated dosing aids for accurate dosing. Then, a post-awareness survey was conducted to re-evaluate their choice of the right oral dosing aid and knowledge about dosing errors using household spoons. This assessment was made to evaluate the effectiveness of this health literacy activity so that it can be recorded if the key information was successfully understood by the respondents or not. The same methodology was repeated to conduct the survey and awareness training for the rest of the eight-degree programs, assisted by the volunteer trainers by conducting in-person surveys and awareness sessions.

Analysis was carried out on the collected data using MS Excel and Graph Pad Prism, Version 8.0.2. The Fischer exact test and the chi-square test were used for the comparison of pre- and post-test results. Recommendations were also designed based on the results and literature survey obtained to improve public health literacy regarding the safe use of medication, especially oral liquid dosage form.

## Results

Pre- and post-intervention surveys were conducted from 118 and 108 participants, respectively. The 10 mismatched respondents that did not appear in the post-test were excluded from the study.

**Figure 3:**
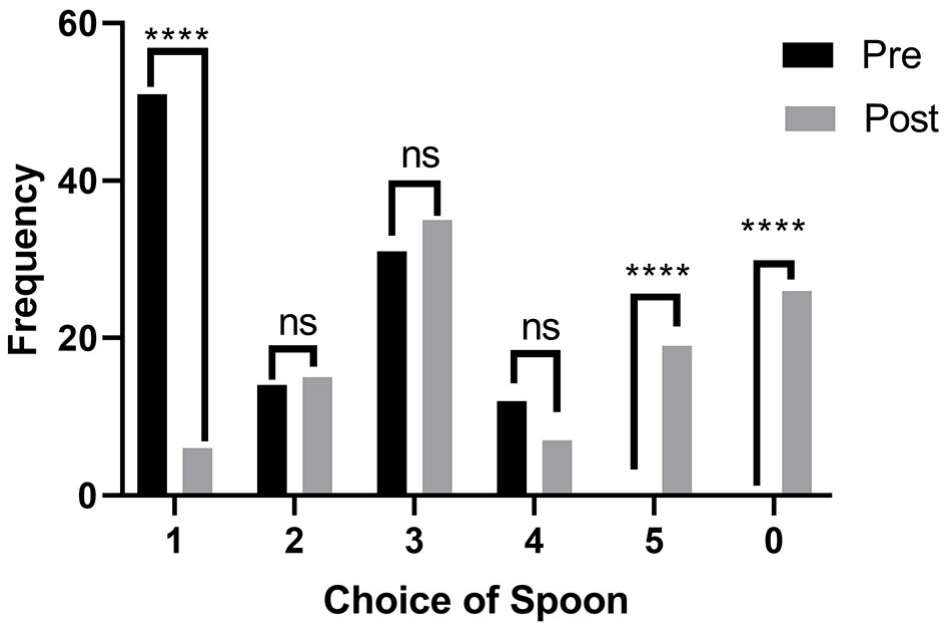
Pre- and post-intervention selection of spoons from household spoons by respondents for oral liquid medicine administration. ^****^means very highly significant.

Figure 3 shows the age distribution of the respondents, falling mainly between 18 and 23 years of age, with 75% of the respondents being women (Table 1). The majority (79) of students (73.15%) were in the age group of 21–24 and had passed their third professional (75%), whereas (28) 25.93% of them were 18–20 years and had passed their second professional exam (25%). Only one student was aged in between 24 and 26 years and was from the fifth semester (Table 1). Eight students did not share their passing years. Most respondents belonged to subjects related to medical sciences, including 30 from Human Nutrition and Dietetics (HND), 21 from Pharm.D., and 15 from Biotechnology and Food Science and Technology (FST). The other degree programs included Microbiology, Medical Laboratory Technology, Environmental Sciences, and Doctor of Veterinary Medicine. The participants were residents of Punjab Province, and 43% were inhabitants of Lahore, with 5.5% from Gujranwala and 4% from Faisalabad.

**Table 1:** Frequency distributions of participants.

**Parameter**	**No. of students (*n* = 108)**	**Percentage (%)**
**Age (years)**		
18–20	28	26
21–23	79	78
24–26	1	1
**Gender**		
Men	27	25
Women	81	75
**Degree program**		
Pharm.D	19	21%
HND	28	30%
FST	14	15%
Biotechnology	14	15%
Microbiology	7	8%
MLT	6	6%
Biological Sciences	5	5%
Environmental Sciences	5	5%
DVM	2	2%
**Home city**		
Lahore	46	43%
Gujranwala	27	25%
Faisalabad	4	4%
Burewala	3	3%
Sialkot	3	3%
Toba Tek Singh	3	3%
Ali Pur Chatta	2	2%
Bahawalnagar	2	2%
Bahawalpur	2	2%
Kasur	2	2%
Okara	2	2%
Sheikhupura	2	2%
Zafarwal	2	2%
Others	5	5%

Pharm.D, Doctor of Pharmacy; HND, Human Nutrition and Dietetics; FST, Food Science and Technology; MLT, Medical Lab Technology; DVM, Doctor of Veterinary Medicine.

In question 1 of the surveys, the respondents were asked to select a household spoon from the set of five spoons (Types I–V) that should be used to administer one spoon of medicine, as shown in Figure 1, without mentioning the volume they can hold. Type I, the largest spoon was chosen by 51 (47.22%) respondents, whereas Type III was the second most popular choice with 31 (28.7%), followed by Type II [14 (12.96%)] and Type IV [12 (11.11%)]. On the contrary, no one chose Type V, the smallest spoon or the option of none in the pre-test. In the post-test, only 6 (5%) respondents chose the Type I spoon; there was an increase of four responses seen for Type III and 19 (17.59%) respondents opted for the smallest spoon, and 26 (24.07%) for the options none/blank (Figure 3). The chi-square test showed that there was a highly significant overall change in the pre- and post-test responses (^****^p < 0.0001). Similarly, the application of the Fischer exact test on individual options for pre- and post-values revealed that a highly significant (^****^p < 0.05) decrease was seen for the choice of type I spoon, whereas there was a highly significant (^****^p < 0.05) increase for choice of spoon type V and the introduction of the choice of none of the spoons (Figure 3). The shift in other options tested through the Fischer exact test was found to be not significant.

**Figure 4:**
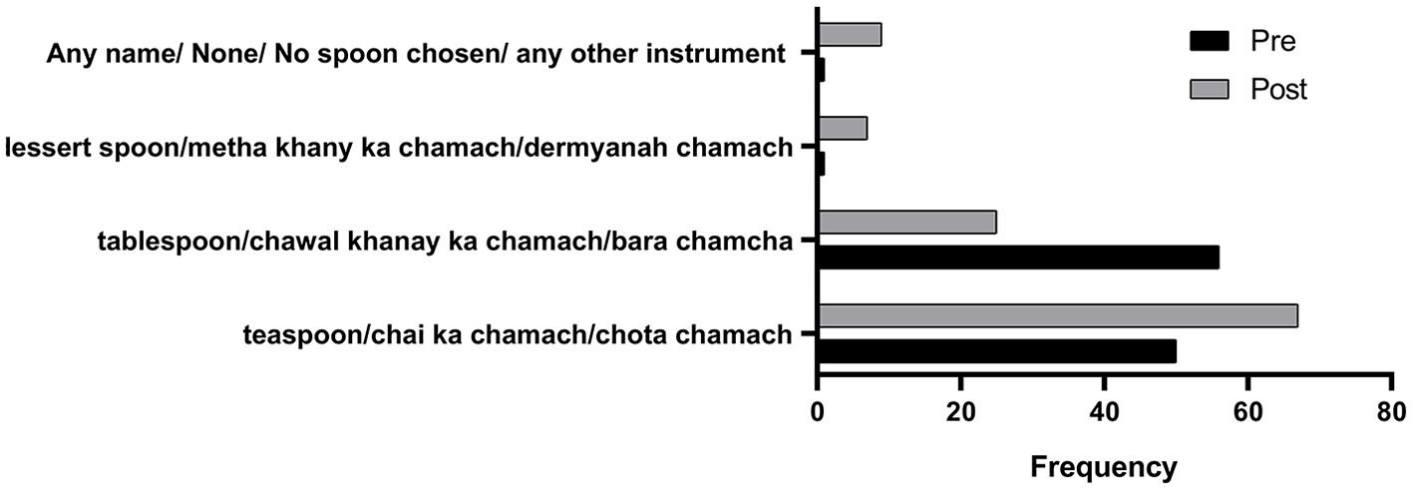
Pre- and post-intervention responses on the name of the spoon chosen for medicine administration. **** means very highly significant.

To confirm the choice of the type of spoon, a second question inquired the respondents about the household name of the spoon they chose in question 1. It was revealed that 56 (46%) respondents intended to choose the option *tablespoon/chawal khanay ka chamachs/bara chamcha* (rice eating spoon/big spoon), whereas 50 (42%) respondents opted for *teaspoon/chai ka chamach/Chota chamach* (Tea Spoon in Urdu/small spoon) as the name of the spoon they would choose for administering medicines. This explains that even the information on medicine spoons being based on teaspoon measures is also faulty in half of the population. In the post-test, the question was rephrased to ask what they would call their chosen spoon in their households after attending the session; the response of 62% of respondents was for the tablespoon option, which confirms that 65 out of 108 (55%) respondents chose the spoon Types I and II in the pre-test (Figure 4). Again, the application of the chi-square test showed a highly significant change in the responses in pre- and post-test results (^*****^p < 0.00001).

**Figure 5:**
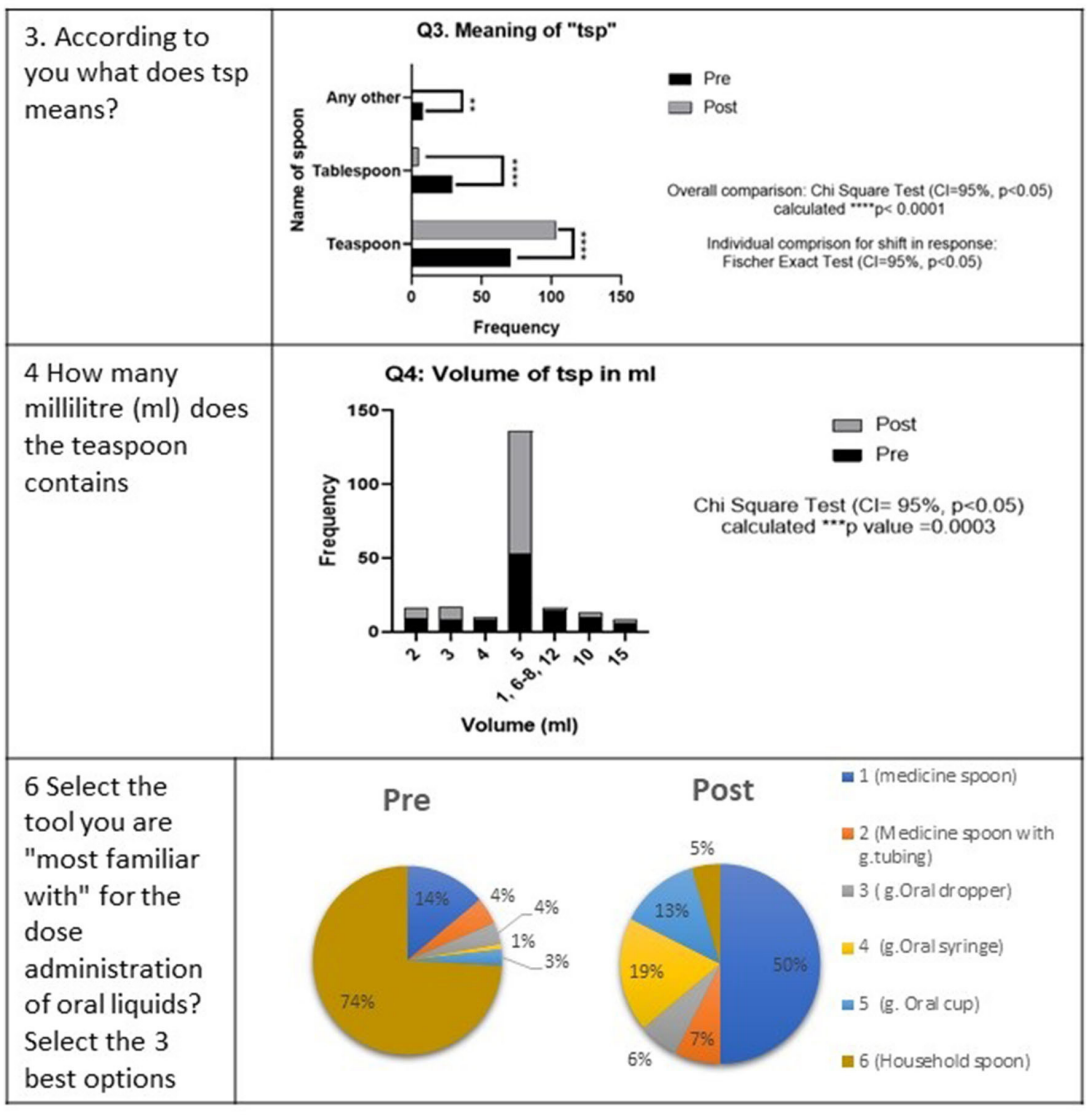
Comparison of pre- and post-intervention responses of the survey participants on the choice of a spoon for medicine administration. ** means very significant. *** means highly significant.

In response to question 3 where respondents were inquired about what they understood from the abbreviation “tsp,” the options were grouped as “teaspoon,” “tablespoon,” and “any other.” A 29.6% gain was observed in the respondents opting for teaspoons as the answer in the post-test. The pre-test result showed that 29 respondents out of 108 opted for the tablespoon option, which was reduced to five in the post-test (Figure 5). The highly significant change was demonstrated by the application of the Fischer exact test on individual change in response (^****^p < 0.05) and an overall change of high significance using the chi-square test (^****^p < 0.0001).

**Figure 6:**
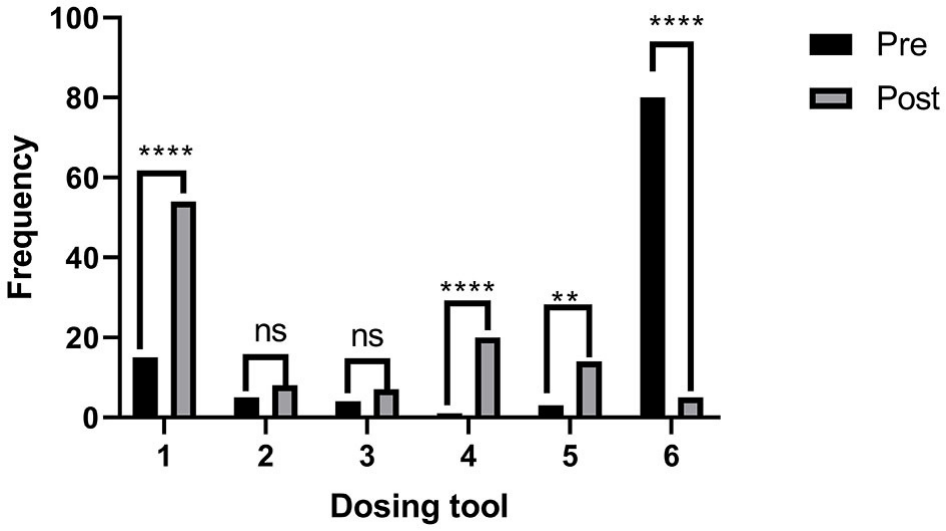
Comparison of pre- and post-intervention responses of the survey participants showing a shift of preference from household spoon to medicine spoon. ^**^means very significant. ^****^means very highly significant.

Similarly, the results were obtained in pre- and post- assessments on the volume in milliliters that a teaspoon holds. A 27.78% increase was observed in the initial number of 53–83 for respondents opting for 5 mL as the volume of a teaspoon (Figure 6). The other popular responses were 7, 10, 2, 3, 4, and 15 mL, with the frequency of 10, 10, 9, 8, 8, and 6, respectively (Figure 5). Whereas, in the post-test results, 2 and 7 mL were the only two other responses above 5% with a frequency of 7 and 9, respectively. The overall change was recorded as high significance using the chi-square test (^***^p = 0.0003).

Respondents were shown the chart carrying photographs of different devices used in the administration of the oral liquid medicines and were asked to pick up the three best options. The selection of household spoons was 74% in the pre-test, which dropped to 5% in the post-test (Figure 5). The order of choices was a household spoon, medicine spoon, oral cup or beaker, cylindrical medicine spoon, graduated dropper, and oral syringe in the pre-test, which changed to a medicine spoon, oral syringe, graduated oral cup/beaker, a medicine spoon with a graduated tube, and graduated oral dropper, followed by the household spoon at the end (Figure 5).

Figure 5 shows the pie chart presentation of pre- and post-responses of the survey participants showing a shift of preference from household spoon to medicine spoon, whereas Figure 6 shows the bar graph presentation with the statistical difference observed in each choice.

**Figure 7:**
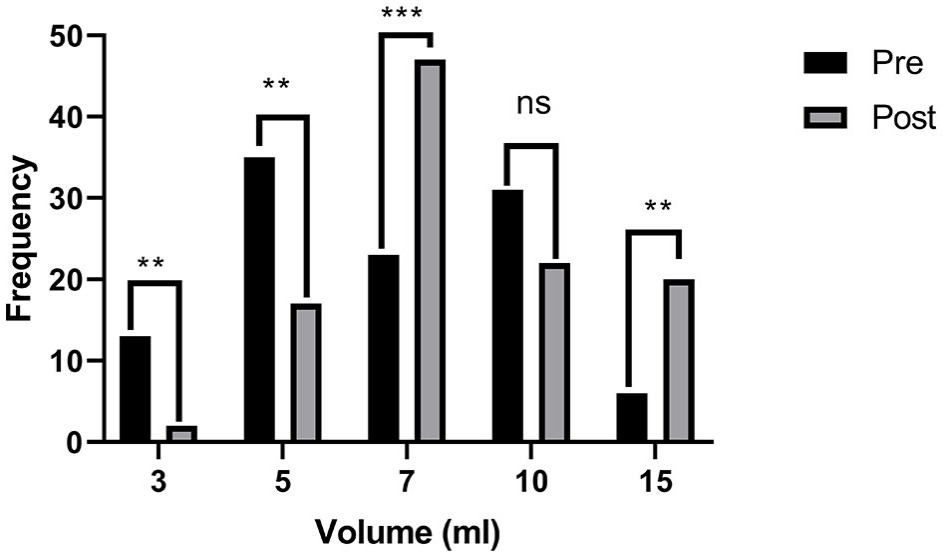
Respondent's answers to the volume of type II spoons are shown in Chart One, pre-, and post-intervention. ** means very significant. *** means highly significant. **** means very highly significant.

Inquiring respondents on the volume of Type II (desert) spoons, the correct responses were significantly increased from 21.3% (pre-test) to 43.5% (post-test) using the Fischer exact test (^**^ to ^***^p < 0.05), except for those who responded it as 10 mL (Figure 7).

**Figure 8:**
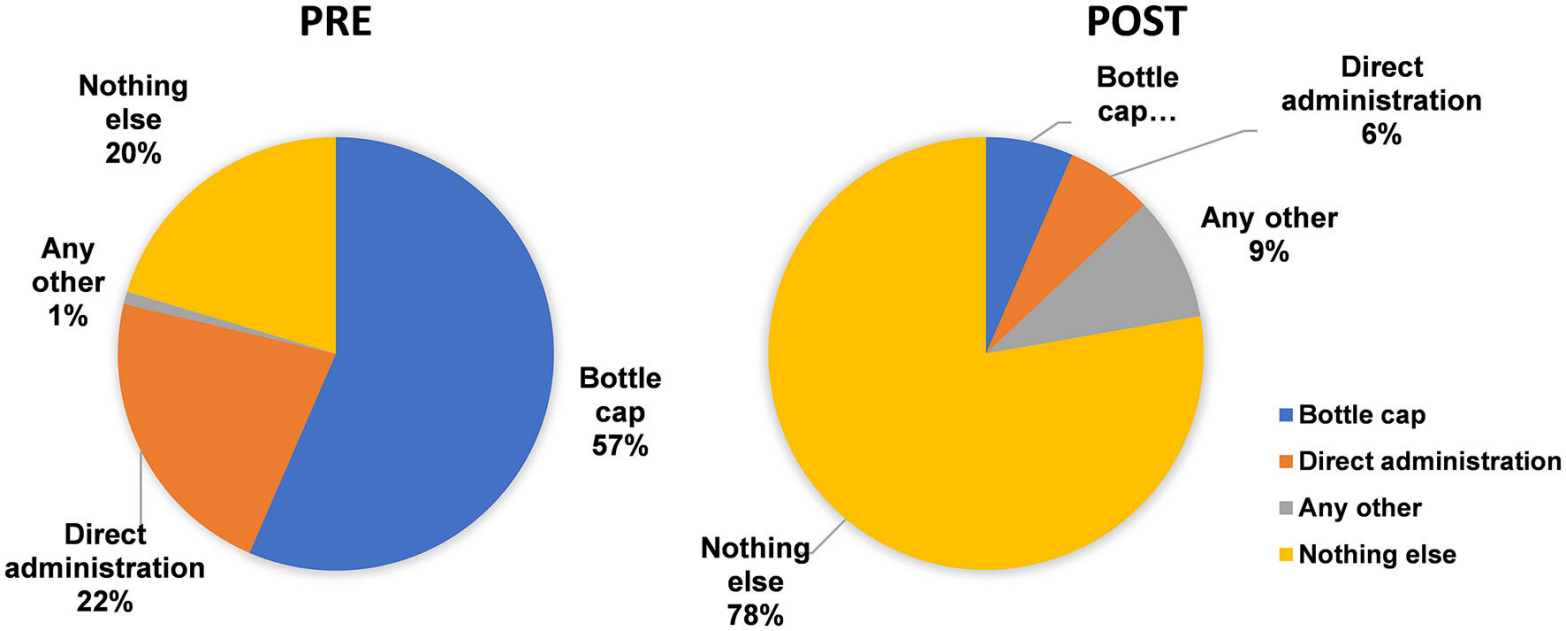
Three best choices for oral medicine administration aids (pre- and post-test).

Another significant shift was observed as the strong contrast appeared in the pre- and post-test for the responses inquiring if they had used any methods other than the shared six methods for dosing oral liquid medicines (Figure 8). During the pre-test, a big proportion of respondents (57%) had used bottle caps, and 22% had admitted direct administration from the bottles. At the same time, this practice was reduced to 7 and 6% in the post-test, respectively. The response that no other method is to be used shifted from 20 to 78% in the post-test.

**Figure 9:**
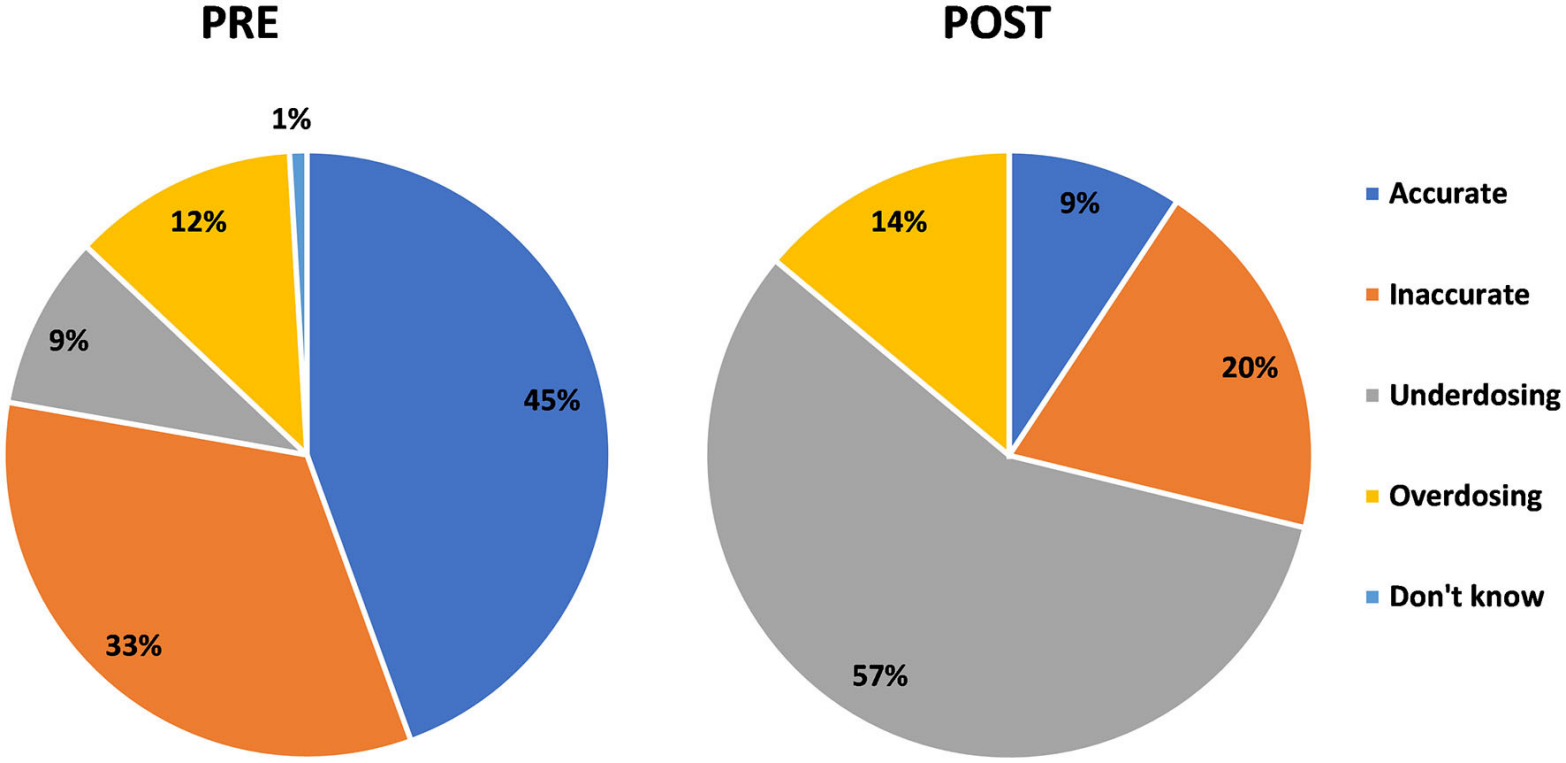
Pre- and post-intervention responses for any other method (than the six tools shared) that can be used for the administration of oral liquid medicines.

Respondents were asked their opinion about their choice of a spoon from chart one if they were to label it as “accurate,” “inaccurate,” “overdosing,” or “under-dosing.” All responses can be assumed as correct except the one given as “accurate,” for which the response was reduced from 45% in the pre-test to 9% in the post-test, which is proof that a good proportion of respondents participating in the study learned about the risk attached to the use of household spoons for the administration of medicines (Figure 9).

**Figure 10:**
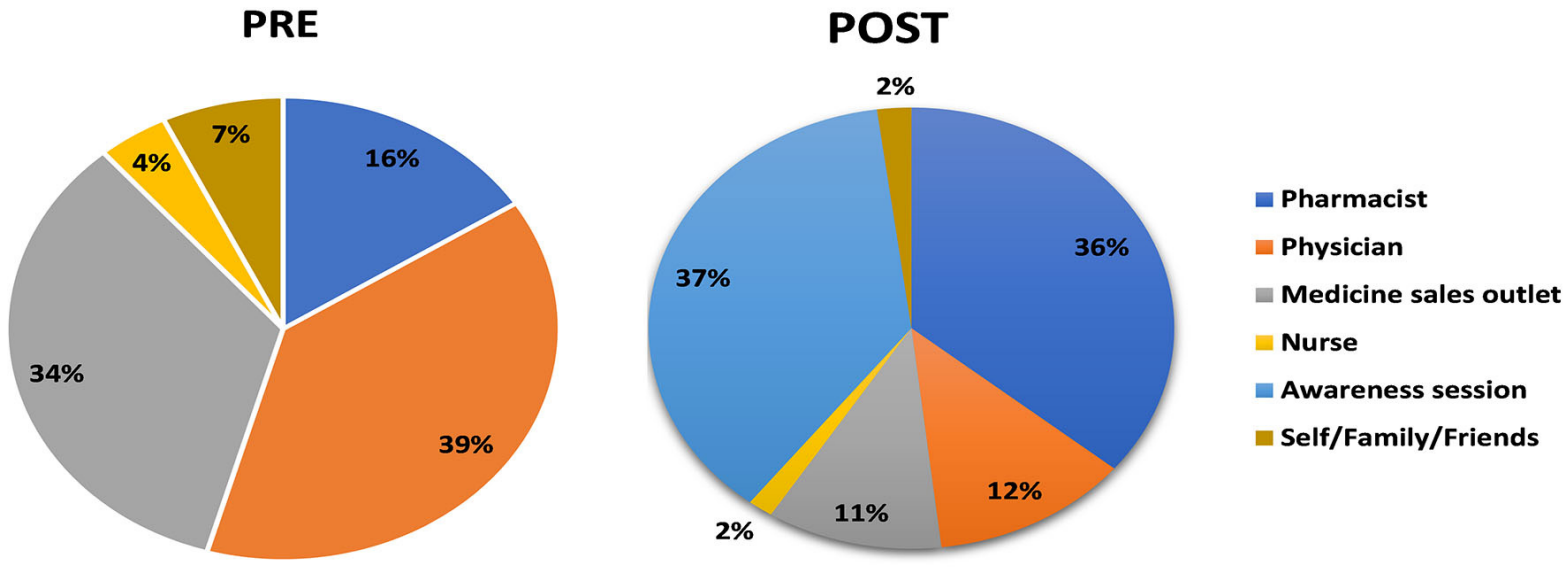
Pre- and post-intervention understanding of the consequence of the use of a household spoon.

In the final question of the survey, respondents were asked to state the information sources from which they learned about the tools for medicine administration. In the pre-intervention assessment, the order of sources from which the respondents obtained information about the selection of tools for medicine administrations was 39% from physicians, 34% from medical stores, 16% by self-understanding, and 7% from family or friends. In the post-intervention assessment, the results shifted to a pharmacist at 36%, an awareness session at 37%, a physician at 12%, and a medical store at 11% (Figure 10).

## Discussion

In the current study, 108 students were successfully assessed for their knowledge and practice on the use of household spoons for administering oral liquid medicines. A household spoon stands universally as the most accessible and commonly used device (5) for the administration of oral liquids, yet it holds the risk of being the most unpredictable one, especially concerning accuracy (12), including mixing tablespoons and teaspoons (5) and handling. Household spoons vary in their design and shape and so may contain varied volumes in different cutlery sets. The same was shown to the respondent during the awareness video (Figure 1), and they were taught the short experiment on how they can measure the volume of the spoons in their household cutlery sets. The current study showed that there is also a mix-up in the understanding of the abbreviation of “tsp” by the respondents as evident from their response to question 3. This inaccuracy was recorded in a study on administering the desired dose of drugs with teaspoons and tablespoons. Teaspoons/tablespoons were collected from 25 households in Attica, Greece. A total of 71 teaspoons and 49 tablespoons were filled with water to measure their capacity. A major portion consisting of 71 teaspoons was recorded to have a volume of 2.5–7.3 mL, making the household teaspoons and tablespoons unreliable as dosing devices, and should no longer be recommended (14).

In the post-test assessment of this study, a sense of consciousness and care was established among the respondents, and they raised a strong opinion of rejecting the use of a household spoon altogether. The awareness session informed the participants on the volume of each spoon. They were informed that none of the spoon is fit for the volume of 5 mL. However, the type III spoon being the closest with 1 mL less than 5 mL and type II being 2 mL in excess of 5 mL. The latter is approximately 0.3 times more than the volume of a medicine spoon (Figure 1). It is interesting to note that the choice of spoon shifted from overdosing (65/108 responses to 21/108 responses) in the post-test, i.e., to under-dosing and recorded the introduction of rejection of the use of a household spoon altogether by choosing none of the spoons (26/108, i.e., 24.07%). The spoons II and I of under-dose choice shifted from a total of 43 (39.81%) to 61 (56.48%). This information brings a caveat for the design of such training activities to simultaneously make an effort to counter the tendency of the respondents to make under-dosing choices. A tendency for under-dosing using a household teaspoon was observed for the acetaminophen (paracetamol) dosing in an Israeli study carried out in 1989 (9). In previous studies, under-dosing has been associated with the ineffectiveness of some therapeutic agents used in emergencies, like paracetamol (9) and ipecac syrup (17), because of the household teaspoons.

In response to question 3, the options were discretely divided into these two choices in the post-test, showing that the respondent got clarity in their opinion; however, there was a small number responding for “tsp” to be interpreted as a tablespoon.

Pre- and post-test assessments on the volume in milliliters that a teaspoon holds showed that only 53 students on the pre-test and 83 students on the post-test opted for the correct choice of 5 mL out of the total 108 students. The incorrect response to the volume of a teaspoon in the 12 and 13th year of education is a serious concern, showing the failure of the education system in imparting basic knowledge to the students. Such a fundamental concept should be a mandatory set of information that any child going through a learning process must receive. Especially, the introduction of the volume units and measures in primary classes can be linked to a practical exercise of calibrating a household teaspoon or cutlery set to make education aimed at improving the understanding of life and its needful skills.

The response regarding the volume of Type II spoons from Chart One also showed (Figure 7) that even the educated population has not been involved in knowing the volume of their household spoons though they have been using them for sensitive tasks like for provision of medicines to the sick ones.

Information on the different oral medicine administration aids showed that respondents were familiar with these aids only to some extent, and the awareness session increased their information. In a previous study, patient caregivers perceived oral beakers/cups as more convenient than oral syringes, which were found to be more accurate in their results. However, the results of the study showed that a big proportion of the caregivers were unable to use both accurately. It also showed that droppers, dosing cups, and household spoons as the most familiar devices for the patients in their settings (12). Another Korean study showed that etched dosing cups, dosing spoons, and printed dosing cups were not so preferred by the public for oral liquid medicine dosing (16). In the current study, the most significant impact was, however, the moving of household spoons from the first place to the last one.

Unsafe practices like using a bottle cap and direct administration from the bottle in the pre-test are suggestive of the lack of proper information on the consequence of wrong doses by the public, even in the educated community. However, little effort led by young professionals through this study changed this negligence into the adoption of awareness and responsibility toward the medication use process. Studies have shown that the correct use of dosing devices was not linked to education, age, or ethnicity (16). Awareness and training sessions are suggested to be essential factors in developing skills regarding these basics in health practices.

The household spoons are not reliable for delivering accurate, safe, and efficacious doses of oral liquid medications. Their reliability is significantly reduced due to improper identification of the spoon type, unavailability, wide variations, spillage, and handling inability. The U.S. Food and Drug Administration (FDA) and National Council for Prescription Drugs in the United States have issued a white paper in which they have also warned that patients should not use their household spoons and choose a more accurate dosing device when taking liquid medications (18, 19). The medicine cups, wells, and cylinders are the most common (80%) dosing devices enclosed with the medicine packs. These devices are comparatively valid and can be relied upon to deliver specific doses of oral liquid medications effectively and efficiently, with correct use. They also possess other benefits, including ease of handling and use, and are less susceptible to spillage (18, 19). The current study also noted that the choice of the household spoon can never be accurate in the respondents' minds.

In addition, efforts should be made to standardize the dosing labeling and instructions (18, 19). To administer most oral liquid prescription medications, a patient or caregiver depends on the prescription container label dosing designations to guide the measurement of the correct dose with a dosing device. The use of multiple volumetric units (e.g., teaspoons, tablespoons, and droppers) and multiple abbreviations (e.g., mL, cc, MLS; tsp, TSP, and t) increases the likelihood of dosing errors by healthcare professionals, patients, and caregivers, which may result in patient harm (20). Milliliters should be the standard unit of measure used on prescription container labels for oral liquid medications (21, 22).

In this study, the response for dose accuracy using a household spoon was reduced from 45% in the pre-test to 9% in the post-test, which is proof that a good proportion of respondents participating in the study learned about the risk attached to the use of household spoons for the administration of medicines and considered it inaccurate, overdosing, or under-dosing. The study was able to register the importance of pharmacists and the use of awareness sessions as a medium for receiving information on the safe and proper use of medicines.

## Conclusion

Knowledge of correct liquid dose measurement tools and volumes of household spoons were assessed successfully, and it was found that there is a deficit in the knowledge of proper use of measuring devices for oral liquid medicines in the educated population, which can be enhanced through simple tools like short video presentations and awareness seminars.

## Recommendations

Based on the results of this study, it is recommended that comprehensive information on the choice of the spoon should be included in the academic and professional curriculum using practical exercises, videos, and graphical presentations to improve the safe administration of oral liquid medicines. Similarly, the regulatory authority should make it a mandatory requirement for the manufacturers to include necessary graphical instructions in the patient information leaflets. It is also recommended that more studies on medicine administration should be conducted in other populations to identify the gaps and propose strategies for the safe use of medicines.

## Limitations

The data were collected only from one country, and all respondents were students from a public sector university. Because of the limited period of the study, reinforcement through multiple interventions and their evaluation of information retention could not be included in the study design.

## Data availability statement

The raw data supporting the conclusions of this article will be made available by the authors, without undue reservation.

## Ethics statement

The studies involving human participants were reviewed and approved by Institutional Review Board of the University of Veterinary and Animal Sciences. The patients/participants provided their written informed consent to participate in this study.

## Author contributions

EY, MF, and AA collected data and made initial draft of manuscript. HN designed figures and illustrations. SA performed statistical analysis of data. MU and HR edited and finalized the manuscript. HR, MP, and AS conceived the idea and designed the study. HR supervised the study. All authors contributed to the article and approved the submitted version.
