# Supplementary material for: Correct administration aid for oral liquid medicines: Is a household spoon the right choice?
Source: Front Public Health. 2023 Feb 20;11:1084667. doi: 10.3389/fpubh.2023.1084667 (PMC9986283; doi:10.3389/fpubh.2023.1084667)
Supplement: Supplementary file 1 [file Data_Sheet_1.PDF]

Supplementary File 1

The right administration aid for oral liquid medicines: Is the household spoon the right choice?

Pre Survey Questions:

1. Your doctor has prescribed you to take 1 spoon of cough syrup twice a day. Choose a spoon you often use from the following chart.
2. What do you call your chosen spoon in your home?
3. According to you what does tsp means?
4. How many milliliter (ml) does the teaspoon contains?
5. How many milli liters (ml) are there in spoon 2?
6. Select the tool you are "most familiar with" and consider best for the dose administration of oral liquids? select the best 3 options
7. Select the dosing tool that you "have mostly used" for the dose administration of oral liquids?
8. How do you get to know about these tools?
9. Any other method which you have used for oral dosing?
10. What do you think the teaspoon you choose in question 1 for oral drug administration can cause?

Post Survey Questions:

1. Your doctor has prescribed you to take 1 spoon of cough syrup twice a day. What you choose now after awareness session.
2. What do you call your chosen spoon in your home?
3. According to you what does the abbreviation word 'tsp' means?
4. After awareness session what do you think how many milliliter (ml) does the teaspoon contains?
5. After orientation session how many milli liters (ml) are there in spoon 2?
6. Select the tool you are "most familiar with" and consider best for the dose administration of oral liquids? select the best 3 options
7. Select the dosing tool that you "have mostly used" for the dose administration of oral liquids?
8. How do you get to know about these tools?
9. After the session, would you like to choose any other method for oral dosing?
10. Please answer this question only after the awareness video is watched and understood. According to the spoon which you choose in pre survey form. What do you think the choice of your dose administration tool can cause_______________?
